# Analysis by Gender and Race and Ethnicity of Reviewers and Awardees for Intramural Research Funding in the Veterans Health Administration

**DOI:** 10.1001/jamanetworkopen.2022.51353

**Published:** 2023-01-18

**Authors:** Taylor L. Boyer, Utibe R. Essien, Terrence M. A. Litam, Leslie R. M. Hausmann, Katie J. Suda

**Affiliations:** 1 Center for Health Equity Research and Promotion, Veterans Affairs Pittsburgh Healthcare System, Pittsburgh, Pennsylvania; 2 Division of General Internal Medicine, University of Pittsburgh School of Medicine, Pittsburgh, Pennsylvania

## Abstract

**Importance:**

Underrepresentation of women and racial and ethnic minority individuals among reviewers and funded investigators in health research has been widely reported. It is unknown whether such underrepresentation exists in the Veterans Health Administration (VHA).

**Objective:**

To describe gender and racial and ethnic makeup of VHA Health Services Research and Development (HSR&D) study sections and funding awardees and to explore the associations between gender and racial and ethnic makeup of study sections and awardees who were women and racial and ethnic minority individuals.

**Design, Setting, and Participants:**

This cross-sectional study at the VHA evaluated study section members and funding awardees from 5 review cycles from March 13, 2018, through March 6, 2020, identified via the HSR&D and National Institutes of Health websites.

**Main Outcomes and Measures:**

Univariable and multivariable logistic regression models were used to determine the likelihood of an awardee being a woman or a racial or ethnic minority individual based on the gender and racial and ethnic makeup of study sections, respectively. Separate models were conducted for investigator-initiated research proposals only.

**Results:**

Thirty-six study sections comprised 664 reviewers, including 381 women (57.4%) and 81 racial and ethnic minority individuals (12.2%). Of the 146 funded proposals, 77 (52.7%) were awarded to women and 25 (17.1%) to racial and ethnic minority investigators; 29 (19.9%) were reviewed by study sections with no racial and ethnic minority reviewers. The odds of a woman awardee were more than 5 times greater for proposals reviewed by study sections comprising the highest proportion of women (fourth quartile; >58.3%) than the lowest proportion of women (first quartile; ≤50.0%) (adjusted odds ratio [aOR], 5.24 [95% CI, 1.70-16.13]). Similarly, the odds of a racial or ethnic minority awardee were 3 times greater for proposals reviewed by study sections in the top 50th percentile for proportion of racial and ethnic minority individuals (>7.3%) than the bottom 50th percentile (aOR, 3.08 [95% CI, 1.18-8.09]). Secondary analyses limited to investigator-initiated research proposals identified similar associations.

**Conclusions and Relevance:**

In this cross-sectional study of VHA HSR&D-funded research, racial and ethnic minority individuals were underrepresented among investigators and reviewers. More women and racial and ethnic minority reviewers on study sections were associated with women and racial and ethnic minority individuals awarded funding.

## Key Points

**Question:**

What is the gender and racial and ethnic makeup of study sections and funding awardees within the Veterans Health Administration, and are there associations between study section and awardee diversity?

**Findings:**

In this cross-sectional study of 664 reviewers and 146 funded proposals, 381 reviewers (57%) and 77 awardees (53%) were women; 81 reviewers (12%) and 25 awardees (17%) were racial and ethnic minority individuals. Having more reviewers on study sections who were women and racial and ethnic minority individuals was positively associated with women and racial and ethnic minority investigators awarded funding.

**Meaning:**

These findings suggest that demographic diversity within study sections is associated with increased funding awarded to women and racial and ethnic minority investigators.

## Teaser

This cross-sectional study explores the representation of women and individuals from racial and ethnic minority groups among Veterans Health Administration Health Services Research and Development study sections and funding awardees.

## Introduction

For over a decade, gender and racial and ethnic disparities among federally funded health research investigators have been widely reported. In 2021, Executive Order 14035 was signed to advance diversity, equity, and inclusion (DEI) within the US government workforce.^1^ In response, the Veterans Health Administration (VHA) Office of Research and Development (ORD) established a DEI workgroup to foster a diverse scientific workforce of different perspectives and backgrounds to address veterans’ complex health problems and stimulate research on health disparities among underserved veterans.^2^ While the diversity of VHA investigators has, to our knowledge, not yet been studied, gender and racial and ethnic disparities among grantees have been reported for the National Institutes of Health (NIH), the leading funder of medical research in the US.^3^

In 2011, Ginther et al^4^ found that African American and/or Black (hereinafter referred to as Black) and Asian principal investigators were less likely to be awarded NIH investigator-initiated research (R01) funding than White investigators during fiscal years 2000 through 2006. Nearly 20 years later and after changes to the NIH review process to address bias and promote diversity,^5^ funding disparities persist.^6,7^ White investigators were more than 1.7 times more likely to receive NIH funding than Black investigators in fiscal years 2011 to 2015.^6^ Previous studies have also found that women are less likely to receive NIH funding than men,^7,8,9^ and Black and Asian women are less likely to receive NIH funding than White women.^10^

Within NIH study sections, women and racial and ethnic minority individuals are also underrepresented relative to the general population in the US.^11^ Women are less likely to be reviewers and chairs and more likely to be temporary members and serve on study sections with lower total funding and proposals awarded.^12^ Furthermore, less than 5% of reviewers (2.4%) on NIH study sections reviewing R01 applications from fiscal years 2011 to 2015 were Black.^6^

The NIH funding mechanisms and application and review processes are comparable to those of the VHA ORD,^13^ which funds health research within the nation’s largest integrated health care system.^14^ Like the NIH, the ORD is organized by different areas of research with 4 primary research services: Biomedical Laboratory Research and Development, Clinical Science Research and Development, Rehabilitation Research and Development, and Health Services Research and Development (HSR&D). The investigator-initiated research (IIR) award is generally considered to be equivalent to the NIH R01 award. Besides the relevance to veteran health, the evaluation of scientific merit determined by peer review is the strongest predictor of funding.^13^

Given the VHA ORD’s commitment to DEI and the HSR&D’s comprehensive plan to promote a diverse scientific workforce and expand equity in health services research,^15^ we explored the representation of women and racial and ethnic minority individuals among VHA HSR&D study sections and funding awardees. Also, we examined the association between the gender and racial and ethnic diversity of study sections and representation of women and racial and ethnic minority individuals among awardees. We hypothesized that gender and racial and ethnic diversity of study sections would be associated with women and racial and ethnic minority investigators awarded funding, respectively.

## Methods

This cross-sectional study followed the Strengthening the Reporting of Observational Studies in Epidemiology (STROBE) reporting guideline. The institutional review board of the Veterans Affairs Pittsburgh Healthcare System approved this study as non–human participant research and therefore waived the need for informed consent owing to the use of publicly available data.

### Data Sources

We analyzed VHA HSR&D data on study section members and funding awardees through 5 review cycles from March 13, 2018, through March 6, 2020. We used data that are publicly available online and by request from the HSR&D,^16,17^ which did not include unfunded applications. For funded proposals, we extracted the awardee’s name, academic degree(s), and affiliated institution(s) and the award’s number, title, and funding or project start date. We also collected the name, academic degree(s), and affiliated institutions for study section members, excluding scientific review officers because they do not score applications. Funding awards were determined by funded proposals posted online by the HSR&D.^16,17^

We linked study sections to proposals using the NIH Research Portfolio Online Reporting Tools Expenditures and Results (RePORTER) module^18^—an online database of federally funded proposals—by querying the database using awardee’s name and proposal title and number. Because RePORTER does not include review cycle data, review cycle was determined using the following logic, which was based on the typical timeline of the VHA funding process. For proposals with a funding or project start date between July and December (eg, September 2018), we estimated the proposal was reviewed during the March review cycle of the same year (eg, 2018). For proposals with a funding or project start date between January and June (eg, February 2019), we estimated the proposal was reviewed during the August review cycle of the prior year (eg, 2018).

### Study Population 

Funding awarded included IIR (3-4 years of support), pilot (1-2 years of support), service-directed research (≤4 years of support), and research career scientist (≤5 years of support) awards. We excluded special-emphasis study sections and corresponding awardees that meet irregularly, including innovation and COVID-19 awards, due to the blinded nature of funding review and involvement of HSR&D leadership in funding decisions. Career development awards, equivalent to NIH K awards, were excluded because this analysis focused on primary funding mechanisms typically awarded to midcareer and senior-career investigators.

### Independent Variables and Covariates

We used methods from previous studies to determine gender^19^ and race and ethnicity.^20^ First, we categorized gender as woman or man based on given name for reviewers and awardees with traditionally feminine or masculine names. For those with ambiguous names, gender was assigned based on pronouns and photographs retrieved via an internet search using the individual’s name, academic degree(s), and institution. To determine race and ethnicity (racial or ethnic minority individual [yes or no]), we used photographs obtained via an internet search in corroboration with surname data from the 2010 Census,^21^ which aggregated the counts of races and ethnicities reported for each surname occurring at least 100 times in the 2010 Census. All participants had either a photograph and/or surname data to inform race and ethnicity assignment. Race and ethnicity were categorized into racial and ethnic minority group (including Asian, Black, Hispanic/Latino, Indigenous peoples, Pacific Islander, and >1 race) or not due to limited diversity among awardees and reviewers. Two analysts (T.L.B. and T.M.A.L.) independently assigned individuals’ gender (>99% agreement) and race and ethnicity (>98% agreement) and jointly reviewed discrepancies to establish consensus.^22^

### Positionality

Because of the importance of carrying out gender and racial and ethnic minority research by members of underrepresented groups, this study was conceptualized and led by a non-Hispanic White woman (K.J.S.) and a Black man (U.R.E.), and a non-Hispanic White woman (L.R.M.H.) served as a key advisor and collaborator. The analysts included 1 Asian man (T.M.A.L.) and 1 transgender individual (T.L.B.).

### Statistical Analysis

We tabulated the number of reviewers and awardees overall and by review cycle. For reviewers and awardees, we calculated the number and percentage of women and racial and ethnic minority individuals overall and by review cycle. We also calculated the mean (SD) and range of the proportion of individuals by gender and racial and ethnic minority group per study section. Study sections with less than 1 proposal awarded were excluded. The proportion of women for each study section was categorized into quartiles and the proportion of racial and ethnic minority individuals was categorized into lower and upper 50th percentiles due to the limited range of data. Univariable and multivariable logistic regression models were used to determine the likelihood of an awardee being a woman or a member of a racial or ethnic minority group based on the gender and racial and ethnic composition of study sections, respectively, as well as proposal type and review cycle to assess trends over time. Separate models were conducted for IIR proposals because, like the R01, the IIR is the primary award mechanism. Sensitivity analyses were conducted using random-effects logistic regression models clustered by study section to account for study section differences (eg, topic, number of proposals and awards). Statistical analyses were performed using Stata Standard Edition, version 17.0 (StataCorp LLC). Significance was assessed at 2-sided *P* < .05.

## Results

### Gender, Race and Ethnicity, and Study Sections

**Figure 1.**
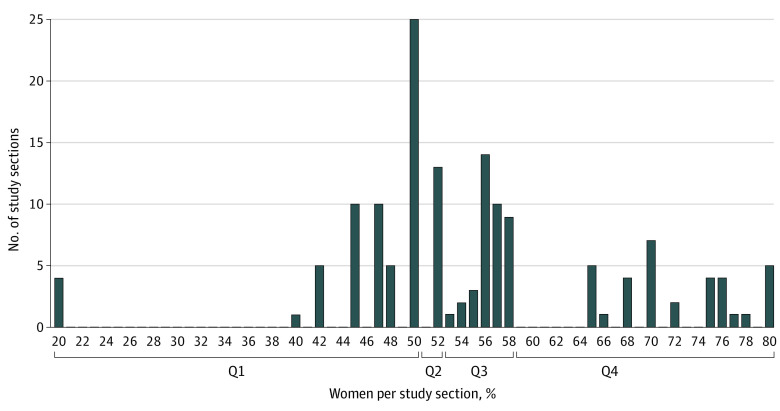
Frequency of Proportions of Women Reviewers on Study Sections for Each Funded Proposal, Grouped by Quartiles Thirty-six unique study sections convened over 5 review cycles; study sections are individually represented for each of the 146 proposals awarded funding.

**Figure 2.**
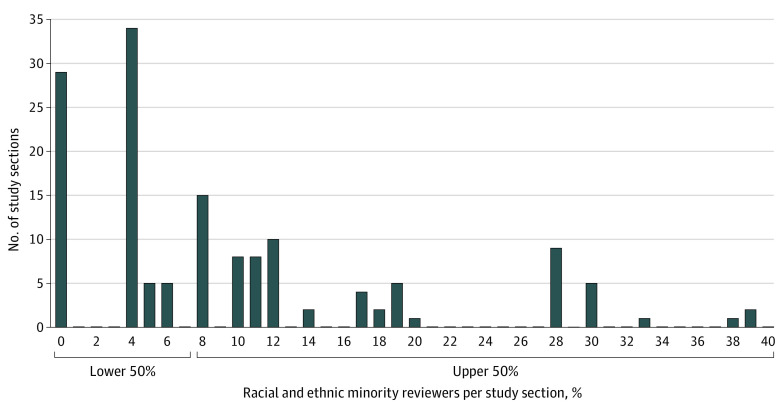
Frequency of Proportions of Racial and Ethnic Reviewers on Study Sections for Each Funded Proposal, Grouped by Percentiles Thirty-six unique study sections convened over 5 review cycles; study sections are individually represented for each of the 146 proposals awarded funding.

Thirty-six study sections convened with 664 reviewers, including 381 women (57.4%) and 81 racial and ethnic minority individuals (12.2%) (Table 1). Per study section, women reviewers ranged from 20.0% to 80.0% (mean [SD], 57.2% [12.6%]). The lower quartile (quartile 1) for the proportion of women was 50.0% or less; the upper quartile (quartile 4), greater than 58.3% (Figure 1). The proportion of racial and ethnic minority reviewers per study section never reached majority standing (range, 0-38.1%; mean [SD], 11.1% [10.9%]). The lower 50th percentile for proportion of racial and ethnic minority reviewers was 7.3% or less; 29 of 146 funded proposals (19.9%) were reviewed by study sections with no racial and ethnic minority reviewers (Figure 2).

**Table 1.** Representation of Women and Racial and Ethnic Minority Individuals Among Reviewers and Awardees, Overall and by Review Cycle

Review cycle	No. of reviewers or awardees	Women, No. (%)	Women per study section, mean (SD) [range]	Racial and ethnic minority individuals, No. (%)	Racial and ethnic minority individuals per study section, mean (SD) [range]
**Reviewers**					
All	664	381 (57.4)	57.2 (12.6) [20.0-80.0]	81 (12.2)	11.1 (10.9) [0-38.1]
March 2018	138	72 (52.2)	48.7 (14.9) [20.0-68.0]	18 (13.0)	11.3 (9.6) [0-28.0]
August 2018	138	77 (55.8)	56.0 (10.5) [40.0-70.6]	17 (12.3)	12.1 (12.9) [0-30.0]
March 2019	139	86 (61.9)	61.4 (11.4) [47.4-78.6]	17 (12.2)	10.6 (12.2) [0-38.1]
August 2019	130	81 (62.3)	63.9 (12.0) [46.8-80.0]	18 (13.8)	12.7 (12.8) [0-34.8]
March 2020	119	65 (54.6)	55.4 (11.6) [42.9-76.5]	11 (9.2)	8.5 (8.1) [0-23.8]
**Awardees**					
All	146	77 (52.7)	56.6 (36.3) [0-100]	25 (17.1)	17.5 (25.4) [0-100]
March 2018	34	22 (64.7)	60.0 (34.6) [0-100]	9 (26.5)	33.6 (33.8) [0-100]
August 2018	28	15 (53.6)	56.1 (44.0) [0-100]	6 (21.4)	27.3 (33.3) [0-100]
March 2019	14	7 (50.0)	50.0 (46.3) [0-100]	2 (14.3)	8.3 (15.4) [0-33.3]
August 2019	29	17 (58.6)	68.2 (28.8) [22.2-100]	4 (13.8)	8.9 (16.0) [0-40.0]
March 2020	41	16 (39.0)	48.3 (27.1) [20.0-100]	4 (9.8)	7.8 (9.8) [0-20.0]

### Gender, Race and Ethnicity, and Funding Awardees

A total of 146 proposals were awarded to 77 women (52.7%) and 25 racial and ethnic minority investigators (17.1%). Awardees per study section included a mean (SD) of 56.6% (36.3%) women and 17.5% (25.4%) racial and ethnic minority individuals. Most investigators received funding for IIR proposals (116 [79.5%]); the remaining investigators were funded for pilot (19 [13.0%]), service-directed research (9 [6.2%]), and research career scientist (2 [1.4%]) awards.

### Awardees and Study Section Diversity

The odds of a woman awardee were greater for proposals reviewed by study sections made up of the highest proportion of women (ie, quartile 4) (odds ratio [OR], 3.50 [95% CI, 1.43-8.57]; *P* = .01) and lower for proposals reviewed in March 2020 (OR, 0.35 [95% CI, 0.14-0.90]; *P* = .03) (Table 2). After adjusting for review cycle and proposal type, the odds of a woman awardee were more than 5 times greater for proposals reviewed by study sections with the highest proportion of women than the lowest proportion (ie, quartile 1) (adjusted OR [aOR], 5.24 [95% CI, 1.79-16.14]; *P* = .004) (OR, 3.09 [95% CI, 1.20-7.93]; *P* = .02). After adjusting for covariates, the odds of a racial or ethnic minority awardee were more than 3 times greater for proposals reviewed by study sections in the top 50th percentile for proportion of racial and ethnic minority reviewers than the bottom 50th percentile (aOR, 3.08 [95% CI, 1.18-8.09]; *P* = .02). Findings were similar when awards were restricted to IIR proposals (Table 3).

**Table 2.** Characteristics Associated With Women and Racial and Ethnic Minority Awardees for All Health Services Research and Development–Funded Proposals, Univariable and Multivariable Logistic Regression Models

Awardee	Univariable		Multivariable	
	OR (95% CI)	*P* value	aOR (95% CI)	*P* value
**Woman **				
Women on study section, %				
Quartile 1	1 [Reference]	NA	1 [Reference]	NA
Quartile 2	2.24 (0.66-7.66)	.20	4.93 (0.98-24.73)	.05
Quartile 3	1.40 (0.62-3.17)	.42	1.66 (0.63-4.36)	.31
Quartile 4	3.50 (1.43-8.57)	.01	5.24 (1.70-16.13)	.004
Review cycle				
March 2018	1 [Reference]	NA	1 [Reference]	NA
August 2018	0.63 (0.23-1.75)	.38	0.28 (0.07-1.11)	.07
March 2019	0.55 (0.15-1.93)	.35	0.32 (0.07-1.37)	.13
August 2019	0.77 (0.28-2.14)	.62	0.49 (0.15-1.61)	.24
March 2020	0.35 (0.14-0.90)	.03	0.40 (0.15-1.08)	.07
Proposal type				
Investigator-initiated research	1 [Reference]	NA	1 [Reference]	NA
Pilot	1.11 (0.42-2.93)	.83	1.02 (0.35-2.97)	.97
Service-directed research	8.00 (0.97-66.02)	.05	7.83 (0.88-69.87)	.07
Research career scientist	1.00 (0.06-16.37)	>.99	0.56 (0.03-10.71)	.70
**Racial or ethnic minority individual**				
Racial and ethnic minority individuals on study section, %				
Bottom 50th percentile	1 [Reference]	NA	1 [Reference]	NA
Top 50th percentile	3.09 (1.20-7.93)	.02	3.08 (1.18-8.09)	.02
Review cycle				
March 2018	1 [Reference]	NA	1 [Reference]	NA
August 2018	0.76 (0.23-2.47)	.65	0.83 (0.24-2.87)	.77
March 2019	0.46 (0.09-2.48)	.37	0.43 (0.08-2.41)	.34
August 2019	0.44 (0.12-1.63)	.22	0.45 (0.12-1.78)	.26
March 2020	0.30 (0.08-1.08)	.07	0.31 (0.08-1.14)	.08
Proposal type				
Investigator-initiated research	1 [Reference]	NA	1 [Reference]	NA
Pilot	1.35 (0.41-4.46)	.63	1.61 (0.44-5.82)	.47

Abbreviations: aOR, adjusted odds ratio; NA, not applicable; OR, odds ratio.

**Table 3.** Characteristics Associated With Women and Racial and Ethnic Minority Awardees for Investigator-Initiated Research Proposals, Univariable and Multivariable Logistic Regression Models

Awardee	Univariable		Multivariable	
	OR (95% CI)	*P* value	aOR (95% CI)	*P* value
**Woman **				
Women on study section, %				
Quartile 1	1 [Reference]	NA	1 [Reference]	NA
Quartile 2	1.58 (0.40-6.19)	.51	2.37 (0.40-14.18)	.34
Quartile 3	1.89 (0.77-4.63)	.16	1.70 (0.62-4.66)	.30
Quartile 4	3.16 (1.13-8.80)	.03	3.71 (1.14-12.15)	.03
Review cycle				
March 2018	1 [Reference]	NA	1 [Reference]	NA
August 2018	0.71 (0.23-2.15)	.54	0.39 (0.09-1.73)	.22
March 2019	0.71 (0.17-2.99)	.64	0.54 (0.12-2.46)	.43
August 2019	0.94 (0.30-2.93)	.92	0.64 (0.18-2.20)	.48
March 2020	0.44 (0.16-1.20)	.11	0.45 (0.16-1.29)	.14
**Racial or ethnic minority individual**				
Racial and ethnic minority individuals on study section, %				
Bottom 50th percentile	1 [Reference]	NA	1 [Reference]	NA
Top 50th percentile	4.72 (1.48-15.08)	.01	4.55 (1.38-15.02)	.01
Review cycle				
March 2018	1 [Reference]	NA	1 [Reference]	NA
August 2018	0.49 (0.13-1.88)	.30	0.62 (0.15-2.52)	.51
March 2019	0.56 (0.10-3.16)	.51	0.50 (0.08-3.01)	.45
August 2019	0.37 (0.09-1.58)	.18	0.51 (0.11-2.35)	.39
March 2020	0.22 (0.05-0.89)	.03	0.23 (0.05-1.00)	.05

Abbreviations: aOR, adjusted odds ratio; NA, not applicable; OR, odds ratio.

### Sensitivity Analysis

Regression models accounting for study section produced similar results with 1 exception (eTables 1 and 2 in Supplement 1). In the multivariable models, the odds of a woman IIR awardee for study sections with the greater proportion of women compared with the lowest proportion were not significant (aOR, 3.51 [95% CI, 0.94-13.07]; *P* = .06).

## Discussion

This cross-sectional investigation of diversity among VHA HSR&D study sections and funded investigators measures the current gender and racial and ethnic makeup of VHA researchers and provides a baseline from which to assess progress of future DEI and workforce diversity initiatives. Although women were adequately represented on most study sections, racial and ethnic minority individuals were underrepresented among reviewers. More important, women and racial and ethnic minority investigators were more likely to be funded by study sections that had higher proportions of women and racial and ethnic minority reviewers.

As this is the first study, to our knowledge, to describe the gender and racial and ethnic composition of VHA funding reviewers and awardees, our findings are best compared with those of the NIH. Women were better represented within the VHA than the NIH: more than half of reviewers and awardees were women, which exceeds the overall proportion of women reviewers on NIH study sections^12^ and women NIH awardees.^7^ Of concern, there was a decline in funding awarded to women in March 2020. Also, racial and ethnic minority individuals were far less represented among VHA study sections and funded investigators. Less than one-quarter of awardees were from racial and ethnic minority backgrounds compared with approximately one-third of NIH awardees.^7^ Furthermore, racial and ethnic minority reviewers accounted for less than 10% of VHA study sections compared with approximately one-fourth of NIH study sections.^6^ Similar to women, there was a declining trend, although not statistically significant, in racial and ethnic minority awardees across review cycles. Required collection and reporting of self-reported gender and race and ethnicity of HSR&D investigators is needed to accurately track diversity among reviewers, applicants, and awardees and measure the ongoing progress of DEI initiatives within the VHA scientific community.

Although we cannot infer causality in this study, increasing the diversity of study sections should be explored as one avenue to increase funding awards to women and racial and ethnic minority researchers, for many reasons. First, multiple aspects of the grant review process contribute to funding disparities, including decision to discuss, impact score assignment, and topic.^6,23^ Ultimately, decision-making processes are strongly influenced by reviewers participating in the decision.^6,24^ Lower funding rates for racial and ethnic minority investigators serve to perpetuate underrepresentation among reviewers because reviewers are typically made up of funded investigators.^25^ This cyclical pattern can be broken at the reviewer level, and this study’s findings show promise that greater proportions of racial and ethnic minority individuals on study sections are associated with more diversity among funding awardees. Nonetheless, further research is needed to understand potential mechanisms of this finding, such as the manifestation of reviewer biases throughout the review process and the perceived innovation and rigor of research topics and methods that may be more likely to be included in studies led by women and racial and ethnic minority researchers. Within the VHA, which is increasingly racially, ethnically, and gender diverse,^26^ it is important that the research community reflect the population it serves to ensure research continues to represent the needs of veterans from these groups and address existent disparities in VHA health care.^27,28,29^

Deeper investigation is needed to explore nuanced aspects of VHA reviewers and awardees associated with disparities identified within NIH^12,23,30^ at the reviewer (eg, role, study section funding capacity) and awardee (eg, career level, research topic/study section capacity) levels. Intersectional research is also needed to understand funding for investigators of multiple racial and ethnic minority backgrounds (eg, women from racial and ethnic minority groups).^10^ While this research can be carried out using publicly available data, research is greatly limited by the sparse and fragmented data currently available from funders.

Increased data transparency from the ORD and HSR&D is necessary to thoroughly investigate funding disparities and measure workforce diversity within the VHA scientific community. The HSR&D provides study section membership lists (reviewer name, role, affiliation) for the 2 most recent review cycles,^16^ success rates (total reviewed, streamlined, and funded) by proposal type for the 6 most recent review cycles,^31^ and current and completed funded proposals (investigator name and affiliation, proposal number, title, funding period, and abstract) dating back to the 1990s.^17^ Notably missing are applicant data and comparisons of funding rates for populations known to be underrepresented in medicine. The HSR&D should consider publishing success rates by gender and race and ethnicity similar to statistics displayed within the NIH Data Book via NIH RePORTER.^32^ It is critical to have actionable data to evaluate progress toward the Executive Order 14035, ORD, and HSR&D mission to create a diverse workforce within VHA research.

Funders should heed the body of evidence that investigator publications and study section assignment at least partially explain funding disparities. For instance, Ginther et al^33^ found that 50% of the funding gap between Black and White NIH investigators was attributed to Black investigators having fewer published reports with fewer coauthors and fewer citations. Systemic biases and structural factors within academia contribute to differential publication rates, collaborative networks, and impact of research conducted by different populations. These factors shape reviewers’ perceptions of innovative and high-quality research proposals and therefore must be part of the process of creating a more equitable research ecosystem. Investment in diverse researchers is also needed, especially at the postdoctoral level when the careers of Black and White researchers diverge. For example, the VHA and other funders have supplemental funding mechanisms for research and leadership development,^15,34,35^ specifically for underrepresented populations to receive experience, training, and mentorship that prepare early-career researchers to apply for research funding. However, these pipeline initiatives alone are not enough. Because racial and ethnic minority investigators have their proposals assigned to study sections with lower award rates,^6,23^ funders must allocate research awards in alignment with topics of interest to racial and ethnic minority investigators. Furthermore, structural-level supports are needed within research institutions and teams (eg, DEI proficiency and inclusion practices) to build inclusive climates that attract, retain, and support the success of racial and ethnic minority trainees. Ultimately, funders must renounce disparate award funding and make bold, progressive actions to develop a diverse research workforce and portfolio.

### Limitations

Our study has important limitations. First, demographic data were not self-reported and may have been misclassified. Transgender and nonbinary or gender-diverse individuals’ identities were not represented in these data. Racial and ethnic minority individuals who present as White and/or have a surname from a different racial or ethnic background could have been misclassified. Second, this analysis was limited to funded awardees; thus, we could not describe the investigators who were not awarded funding nor analyze the association between study section diversity and the demographic makeup of investigators of unsuccessful funding applications. Further investigation of applicants is needed to explore demographic differences among investigators of proposals successful at various stages in the review process (eg, proposals scored and discussed vs proposals scored but not discussed). Third, review cycle was inferred from HSR&D-reported start dates and assumed to follow the standard timeline, although the study section that reviewed the application was known. Review cycle may have been inaccurately categorized for proposals with postponed start dates. Additionally, these findings are specific to VHA ORD research services and may not be generalizable to other VHA services or non-VHA research institutes.

## Conclusions

The findings of this cross-sectional study suggest that VHA HSR&D-funded investigators and reviewers from racial and ethnic minority backgrounds are severely underrepresented. The positive association between diversity of study sections and HSR&D-funded women and racial and ethnic minority investigators shows a potential first step to foster diversity among funded investigators. Increased data transparency is needed to thoroughly explore funding disparities and evaluate current and future DEI efforts. Ultimately, bold actions are needed to increase racial and ethnic diversity among VHA HSR&D investigators.

## Notes

Supplement 1. **eTable 1.** Characteristics Associated With Women and Racial and Ethnic Minority Awardees for All Health Services Research and Development–Funded Proposals, Univariable and Multivariable Logistic Regression Models Clustered by Study Section **eTable 2.** Characteristics Associated With Women and Racial and Ethnic Minority Awardees for Investigator-Initiated Research Proposals, Univariable and Multivariable Logistic Regression Models Clustered by Study Section

Supplement 2.
Data Sharing Statement
