# Supplementary material for: Analysis by Gender and Race and Ethnicity of Reviewers and Awardees for Intramural Research Funding in the Veterans Health Administration
Source: JAMA Netw Open. 2023 Jan 18;6(1):e2251353. doi: 10.1001/jamanetworkopen.2022.51353 (PMC9857161; doi:10.1001/jamanetworkopen.2022.51353)
Supplement: Supplement 1. — eTable 1. Characteristics Associated With Women and Racial and Ethnic Minority Awardees for All Health Services Research and Development–Funded Proposals, Univariable and Multivariable Logistic Regression Models Clustered by Study Section eTable 2. Characteristics Associated With Women and Racial and Ethnic Minority Awardees for Investigator-Initiated Research Proposals, Univariable and Multivariable Logistic Regression Models Clustered by Study Section [file jamanetwopen-e2251353-s001.pdf]

Supplementary Online Content

Boyer TL, Essien UR, Litam TMA, Hausmann LRM, Suda KJ. Analysis by gender and race and ethnicity of reviewers and awardees for intramural research funding in the Veterans Health Administration. *JAMA Netw Open*. 2023;6(1):e2251353. doi:10.1001/jamanetworkopen.2022.51353

eTable 1. Characteristics Associated With Women and Racial and Ethnic Minority Awardees for All Health Services Research and Development–Funded Proposals, Univariable and Multivariable Logistic Regression Models Clustered by Study Section

eTable 2. Characteristics Associated With Women and Racial and Ethnic Minority Awardees for Investigator-Initiated Research Proposals, Univariable and Multivariable Logistic Regression Models Clustered by Study Section

This supplementary material has been provided by the authors to give readers additional information about their work.

© 2023 Boyer TL et al. *JAMA Network Open*.

eTable 1. Characteristics Associated With Women and Racial and Ethnic Minority Awardees for All Health Services Research and Development–Funded Proposals, Univariable and Multivariable Logistic Regression Models Clustered by Study Section

	Univariable			Multivariable		
	OR	(95% CI)	P value	aOR	(95% CI)	*P* value
	Woman					
Women on Study Section, %						
Quartile 1	Reference			Reference		
Quartile 2	1.61	(0.41-6.26)	0.49	4.15	(0.64-27.06)	0.14
Quartile 3	1.13	(0.43-2.97)	0.81	1.49	(0.46-4.82)	0.51
Quartile 4	2.73	(0.85-8.84)	0.09	**4.68**	(1.20-18.35)	0.03
Review Cycle						
March 2018	Reference			Reference		
August 2018	0.54	(0.18-1.61)	0.27	0.29	(0.07-1.16)	0.08
March 2019	0.52	(0.13-2.02)	0.35	0.33	(0.08-1.48)	0.15
August 2019	0.82	(0.28-2.45)	0.73	0.52	(0.15-1.83)	0.31
March 2020	**0.35**	(0.12-0.97)	0.04	0.38	(0.14-1.07)	0.07
Proposal Type						
Investigator-initiated research	Reference			Reference		
Pilot	1.04	(0.38-2.89)	0.94	1.01	(0.34-2.96)	0.99
Service-directed research	7.15	(0.76-67.39)	0.09	7.24	(0.76-68.46)	0.08
Research career scientist	0.71	(0.03-16.80)	0.83	0.54	(0.03-11.09)	0.69
	Racial or Ethnic Minority Individual					
Racial and Ethnic Minority Individuals on Study Section, %						
Bottom 50th Percentile	Reference			Reference		
Top 50th Percentile	**2.94**	(1.03-8.37)	0.04	**2.98**	(1.02-8.74)	0.05
Review Cycle						
March 2018	Reference			Reference		
August 2018	0.84	(0.24-2.90)	0.78	0.89	(0.25-3.21)	0.85
March 2019	0.44	(0.08-2.51)	0.36	0.43	(0.07-2.43)	0.34
August 2019	0.40	(0.10-1.54)	0.18	0.45	(0.11-1.82)	0.26
March 2020	0.34	(0.09-1.28)	0.11	0.32	(0.08-1.24)	0.10
Proposal Type						
Investigator-initiated research	Reference			Reference		
Pilot	1.54	(0.44-5.40)	0.50	1.76	(0.47-6.65)	0.41

Note. OR=odds ratio; CI=confidence interval; aOR=adjusted odds ratio; IIR=investigator-initiated research (R01 equivalent); SDR=service directed research; RCS=research career scientist.

© 2023 Boyer TL et al. *JAMA Network Open*.

eTable 2. Characteristics Associated With Women and Racial and Ethnic Minority Awardees for Investigator-Initiated Research Proposals, Univariable and Multivariable Logistic Regression Models Clustered by Study Section

	Univariable			Multivariable		
	OR	(95% CI)	P value	aOR	(95% CI)	*P* value
	Woman					
Women on Study Section, %						
Quartile 1	Reference			Reference		
Quartile 2	1.41	(0.29-6.82)	0.67	1.92	(0.24-15.21)	0.54
Quartile 3	1.84	(0.71-4.74)	0.21	1.60	(0.53-4.85)	0.41
Quartile 4	**3.10**	(1.04-9.27)	0.04	3.51	(0.94-13.07)	0.06
Review Cycle						
March 2018	Reference			Reference		
August 2018	0.60	(0.18-1.93)	0.39	0.40	(0.09-1.82)	0.24
March 2019	0.74	(0.17-3.30)	0.69	0.58	(0.12-2.75)	0.49
August 2019	0.94	(0.30-3.07)	0.93	0.65	(0.18-2.33)	0.51
March 2020	0.41	(0.16-1.20)	0.10	0.43	(0.15-1.27)	0.13
	Racial or Ethnic Minority Individual					
Racial and Ethnic Minority Individuals on Study Section, %						
Bottom 50th Percentile	Reference			Reference		
Top 50th Percentile	**4.72**	(1.48-15.08)	0.01	**4.55**	(1.38-15.02)	0.01
Review Cycle						
March 2018	Reference			Reference		
August 2018	0.54	(0.13-2.25)	0.40	0.62	(0.15-2.52)	0.51
March 2019	0.48	(0.08-2.95)	0.43	0.50	(0.08-3.01)	0.45
August 2019	0.35	(0.08-1.59)	0.18	0.51	(0.11-2.35)	0.39
March 2020	0.23	(0.05-1.03)	0.06	**0.23**	(0.05-0.99)	0.05

Note. OR=odds ratio; CI=confidence interval; aOR=adjusted odds ratio.

© 2023 Boyer TL et al. *JAMA Network Open*.
