# Supplementary material for: Analysis by Gender and Race and Ethnicity of Reviewers and Awardees for Intramural Research Funding in the Veterans Health Administration
Source: JAMA Netw Open. 2023 Jan 18;6(1):e2251353. doi: 10.1001/jamanetworkopen.2022.51353 (PMC9857161; doi:10.1001/jamanetworkopen.2022.51353)
Supplement: Supplement 2. — Data Sharing Statement [file jamanetwopen-e2251353-s002.pdf]

Data Sharing Statement

Boyer. Analysis by Gender and Race and Ethnicity of Reviewers and Awardees for Intramural Research Funding in the Veterans Health Administration. *JAMA Netw Open*. Published January 18, 2023. doi:10.1001/jamanetworkopen.2022.51353

Data
Data available: No
